# Tumor-associated macrophages-derived exosomes promote the migration of gastric cancer cells by transfer of functional Apolipoprotein E

**DOI:** 10.1038/s41419-018-0465-5

**Published:** 2018-03-22

**Authors:** Peiming Zheng, Qin Luo, Weiwei Wang, Junhua Li, Tingting Wang, Ping Wang, Lei Chen, Peng Zhang, Hui Chen, Yi Liu, Ping Dong, Guohua Xie, Yanhui Ma, Li Jiang, Xiangliang Yuan, Lisong Shen

**Affiliations:** 1 Department of Clinical Laboratory, Xinhua Hospital, Shanghai Jiao Tong University School of Medicine, 200092 Shanghai, China; 2 Department of Clinical Laboratory, Henan Provincial People’s Hospital, Zhengzhou, 450000 Henan China; 3 Department of General Surgery, Xinhua Hospital, Shanghai Jiao Tong University School of Medicine, 200092 Shanghai, China; 4 Department of Gynecology, Xinhua Hospital, Shanghai Jiao Tong University School of Medicine, 200092 Shanghai, China

## Abstract

Tumor-associated macrophages (TAMs) are a major component of the tumor microenvironment and have been shown to contribute to tumor aggressiveness. However, the detailed mechanisms underlying the pro-metastatic effect of TAMs on gastric cancer are not clearly defined. Here, we show that TAMs are enriched in gastric cancer. TAMs are characterized by M2-polarized phenotype and promote migration of gastric cancer cells in vitro and in vivo. Furthermore, we find that M2-derived exosomes determine the TAMs-mediated pro-migratory activity. Using mass spectrometry, we identify that apolipoprotein E (ApoE) is highly specific and effective protein in M2 macrophages-derived exosomes. Moreover, TAMs are uniquely immune cells population expressed ApoE in gastric cancer microenvironment. However, exosomes derived from M2 macrophages of *Apoe*^*−/−*^ mice have no significant effect on the migration of gastric cancer cells in vitro and in vivo. Mechanistically, M2 macrophage-derived exosomes mediate an intercellular transfer of ApoE-activating PI3K-Akt signaling pathway in recipient gastric cancer cells to remodel the cytoskeleton-supporting migration. Collectively, our findings signify that the exosome-mediated transfer of functional ApoE protein from TAMs to the tumor cells promotes the migration of gastric cancer cells.

## Introduction

Tumor microenvironment (TME) has a critical role in tumor progression and metastasis^1^. Although the TME comprises a variety of nonmalignant stromal cell types, tumor-associated macrophages (TAMs) are the major constituent immune cells of the TME in many cancers. Growing evidence from clinical and epidemiological studies has shown a strong association between TAM density and poor prognosis in several types of cancer^2–4^, including gastric cancer (GC)^5^. GC is commonly diagnosed and is the top five leading cause of cancer death among both men and women in China^6^. Despite the success of modern chemotherapy and surgical method in the treatment of early-stage cancers^7,8^, patients with metastatic GC continues to have a dismal outcome.

A plethora of elegant studies focusing on TAMs have shown that TAMs are associated with tumor progression and metastasis through intercellular communication with cancer cells, however, research into the communication between TAMs and tumor cells has been limited to soluble factors, such as proinflammatory cytokines, including chemokines, inflammatory factors, and growth factors^2,5^. Recent evidence suggests that exosomes are a vital communication medium between different cell types in the TME^9,10^. Exosomes carry information from one cell to another and reprogram the recipient cells^11^. Most of the current studies are focused on cancer cell-secreted exosomes^12–14^, and little is known about TAM-derived exosomes and their influence on cancer cells. TAMs have two opposing phenotypes; macrophages exhibiting pro-tumorigenic activity are termed M2-type macrophages, in contrast to the antitumorigenic M1 subtype. The phenotype of TAMs is regulated by specific tumor-derived chemokines and exosomes. A recent study has suggested that tumor-derived exosomal miRNA regulates the polarization of tumor-promoting M2 macrophages^15^. However, the regulation of tumor progression and metastasis by TAM-derived exosomes is not clearly defined. Currently, the exosomal profile of TAMs remains largely unknown, and it is unclear whether there are exclusively TAM-derived exosomes that are functionally essential for tumor progression.

In this study, we sought to determine the effect of TAM-derived exosomes on the migration of GC. We found that gastric TAMs were primarily macrophage subpopulation with M2 phenotype. Specifically, we found that M2 exosomes promoted migration of GC in vitro and in vivo. Interestingly, ApoE, an M2-specific and highly rich protein derived from M2 exosomes, was a central driver in determining the migration potential of GC cells.

## Results

### Macrophages are enriched in the TME of human GC and characterized by M2-polarized phenotype

**Fig. 1:**
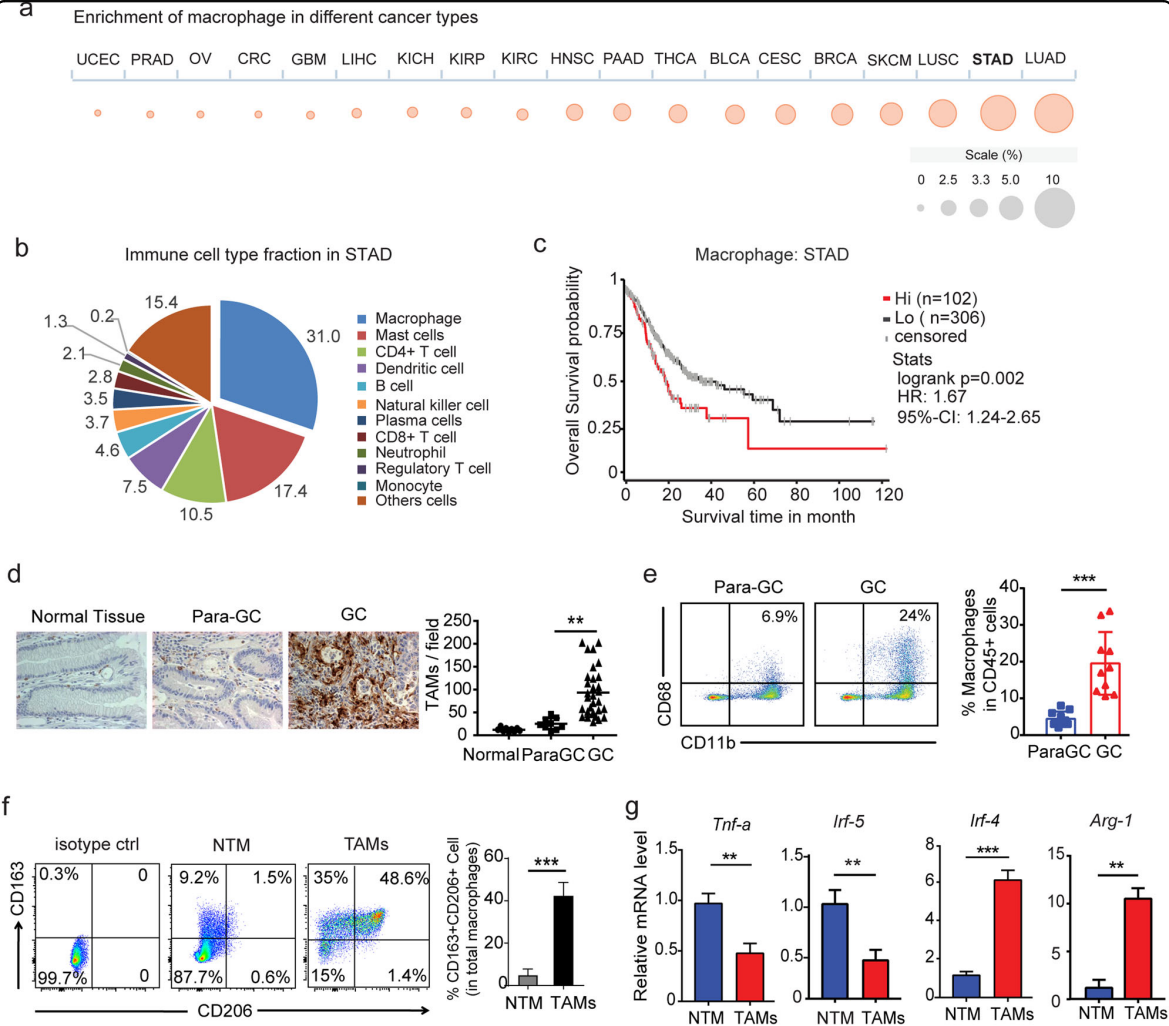
Accumulation of M2-subtype TAMs correlates with aggressive progression of GC. **a** Enrichment of macrophage across 19 solid cancers. Bubble plot shows the results from ssGSEA, where the size of the circles gives the percentage of patients with NES > 0 and *q* value (FDR) < 0.1. **b** The mean fraction of immune subpopulations in STAD (*n* = 142). **c** Overall survival probability of macrophage in STAD (hi: STAD patients with higher macrophage *n* = 102, median OS = 19.3 mons; lo: STAD patients with lower macrophage *n* = 306, median OS = 42.5 mons). **d** IHC analysis and quantification of CD68 expression in the human normal gastric mucosa, para-GC, and GC tissues. Scale bar, 100 μm. **e** FACS analysis and quantification of CD11b^+^CD68^+^ macrophages in human paired GC tissues and adjacent tissues (*n* = 10). **f** FACS analyses of CD163 and CD206 in tissue-resident macrophages (NTM) and TAMs. Dot plots represent three independent experiments with similar results. **g** Gene expression of *tnf-α*, irf-4, irf5, *arg-1* in NTM and TAMs of GC tissues (*n* = 10). *Gapdh* was used as a control. Error bars represent mean ± s.d.; **P < 0.01; ***P < 0.001; n.s. not significant; by unpaired two-sided Student’s *t*-test (**d**, **e**, **f**, **g**)

To evaluate the distribution of macrophages in the TME, we firstly utilized gene set enrichment analysis (GSEA) via the cancer immunome database (TCIA)^16^. The results of the characterization of macrophages showed the heterogeneity across 19 solid cancers, and macrophages were enriched in stomach adenocarcinoma (STAD) (Fig. 1a). The infiltration of immune cellular profiles demonstrated that macrophages were dominant in the TME of GC (Fig. 1b). Moreover, the infiltration of macrophages was indicative of poor clinical outcomes (Fig. 1c). To validate the distribution of macrophages in the TME of GC, we analyzed the expression of CD68, a macrophage marker, by immunohistochemistry (IHC) in human GC tissues of our cohort. We found the higher density of TAMs in cancer tissues than that in para-cancer tissues or normal gastric tissues (Fig. 1d). Flow cytometric quantification also confirmed that the enrichment of macrophages (CD11b^+^CD68^+^) in GC tissues (Fig. 1e). On the basis of the expression of polarization markers, TAMs in human GC was verified as pro-tumor phenotype, characterized by increased M2-associated markers (CD163, CD206) (Fig. 1f, Supplementary Figure S1a, b). The mRNA expression of prototypical M2 markers (Irf4, Arg1) was increased, while the expression of M1 markers (Irf5, Tnfa) was reduced (Fig. 1g, Supplementary Figure S1c). A similar phenotype and gene expression profile were observed in TAMs from the mouse GC model (Supplementary Figure S1d, e).

### Enriched TAMs are associated with metastasis status and M2-polarized macrophages promote the migration of GC cells

**Fig. 2:**
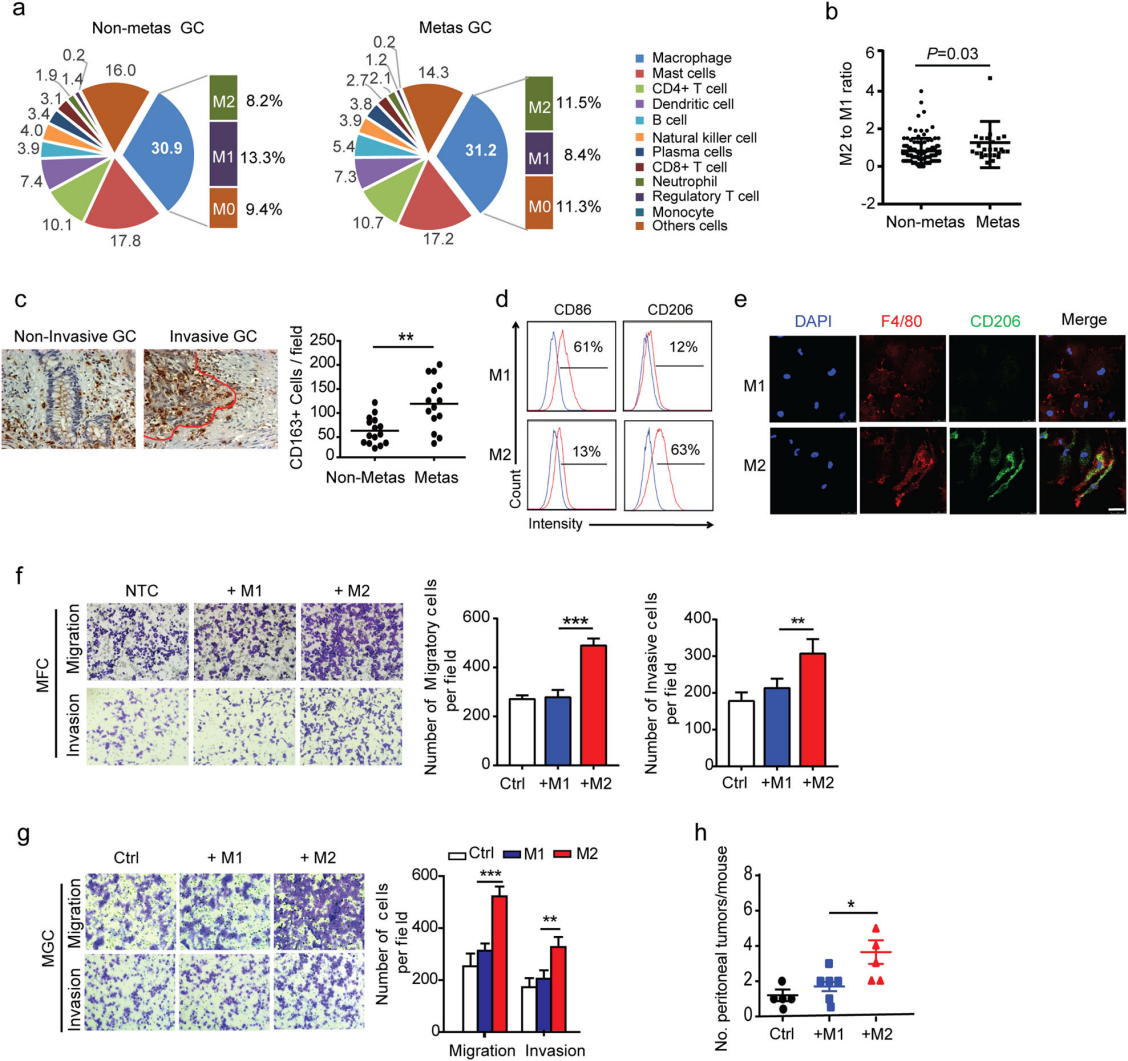
M2-polarized macrophages induce aggressive behavior of human and mouse GC cells. **a** The pie chart shows the mean fraction of immune subpopulations in STAD without (non-metas, *n* = 115) or with metastasis (metas, *n* = 26). The column chart shows the fraction of macrophage subpopulations (M0, M1, and M2) in the macrophage. **b** Quantification of M2/M1 ratio in non-metastatic (*n* = 115) and metastatic STAD (*n* = 26), based on TCIA database. **c** IHC staining and quantification of CD163 expression (M2 marker) in human GC tissues of patients with or without metastasis from our cohort-I (*n* = 15). **d** Flow analysis shows the specific markers of murine bone marrow-derived macrophages (BMDMs) differentiated to M1-polarized (M1) or M2-polarized macrophages (M2). Histograms represent two independent experiments with similar results. **e** Immunofluorescence staining for F4/80 and CD206 in M1 or M2-polarized macrophages from BMDMs. Scale bar represents 25 μm. **f** Migration and invasion assay of mouse GC cells (MFC) cocultured with M1 or M2 macrophages. Shown is the mean ± s.e.m. of two independent experiments. **g** Migration and invasion assay of human GC cells (MGC) cocultured with M1 or M2 macrophages. Shown is the mean ± s.e.m. of two independent experiments. **h** Quantitative analysis of peritoneal metastasis in mice after inoculation with MFC cancer cells educated with M1 or M2-polarized macrophages (*n* = 10 mice in each group). Error bars represent mean ± s.e.m.; **P < 0.01; ***P < 0.001; n.s. not significant; by one-way analysis of variance (ANOVA) with Tukey’s method for multiple comparisons (**b**, **c**, **f**, **g**, **h**)

Using the GSEA strategy, we estimated the three subpopulations of macrophages including macrophage M0, M1, and M2 (Fig. 2a) in STAD patients. The results of the cellular characterization of the macrophage showed that pro-tumoral M2 macrophages were more enriched in metastatic STAD patients than those without metastasis (Fig. 2a, b). The IHC results from our validated cohort also displayed that the majority of the CD163^+^ M2-type TAMs were distributed along the invasive margin (Fig. 2c). There were significantly higher numbers of TAMs in patients with lymph node or distant metastasis (Fig. 2c).

Given that the infiltrating TAMs in GC are primarily M2 macrophages, we wondered if macrophages can be polarized to M2 macrophages by the factors of GC cells. Indeed, our data showed that tumor explant supernatant from MFC xenograft mice-polarized naive macrophages to M2 macrophage in vitro (Supplementary Figure S2a). To determine the functional biology of polarized macrophages, we generated M1/M2-polarized macrophages in vitro from mouse bone marrow cells or human monocytes (Supplementary Figure S2b). The generated M2-polarized macrophages expressed higher levels of the mannose receptor CD206, but reduced levels of CD86 (Fig. 2d, e). The mRNA and cytokine profiles of M2-polarized macrophages were also analogous to those in TAMs (Supplementary Figures S2c, d). We subsequently demonstrated that M2 macrophages significantly promoted more MFC cell migration and invasion (Fig. 2f) with no significant effect on proliferation (Supplementary Figure S2e). A similar observation regarding phenotype profile was made in human M2-polarized macrophages (Supplementary Figure S2f), and M2-polarized macrophages also promoted the migration of human GC cell line (MGC-803) (Fig. 2g), confirming a common function of M2 macrophages in human and mouse models. To assess the effect of M2-polarized macrophages on tumorigenesis in vivo, MFC cells subjected to long-term treatment with different polarization-type macrophages were inoculated into the peritoneal cavity of syngeneic 615 mice. Our results showed that M2 macrophages co-culturing promoted GC aggressiveness by significantly increasing metastasis (Fig. 2h). Taken together, these data suggest that M2-polarized macrophages can promote the migration potential of GC cells.

### M2 macrophage-derived exosomes account for promoting the migration of GC cells

**Fig. 3:**
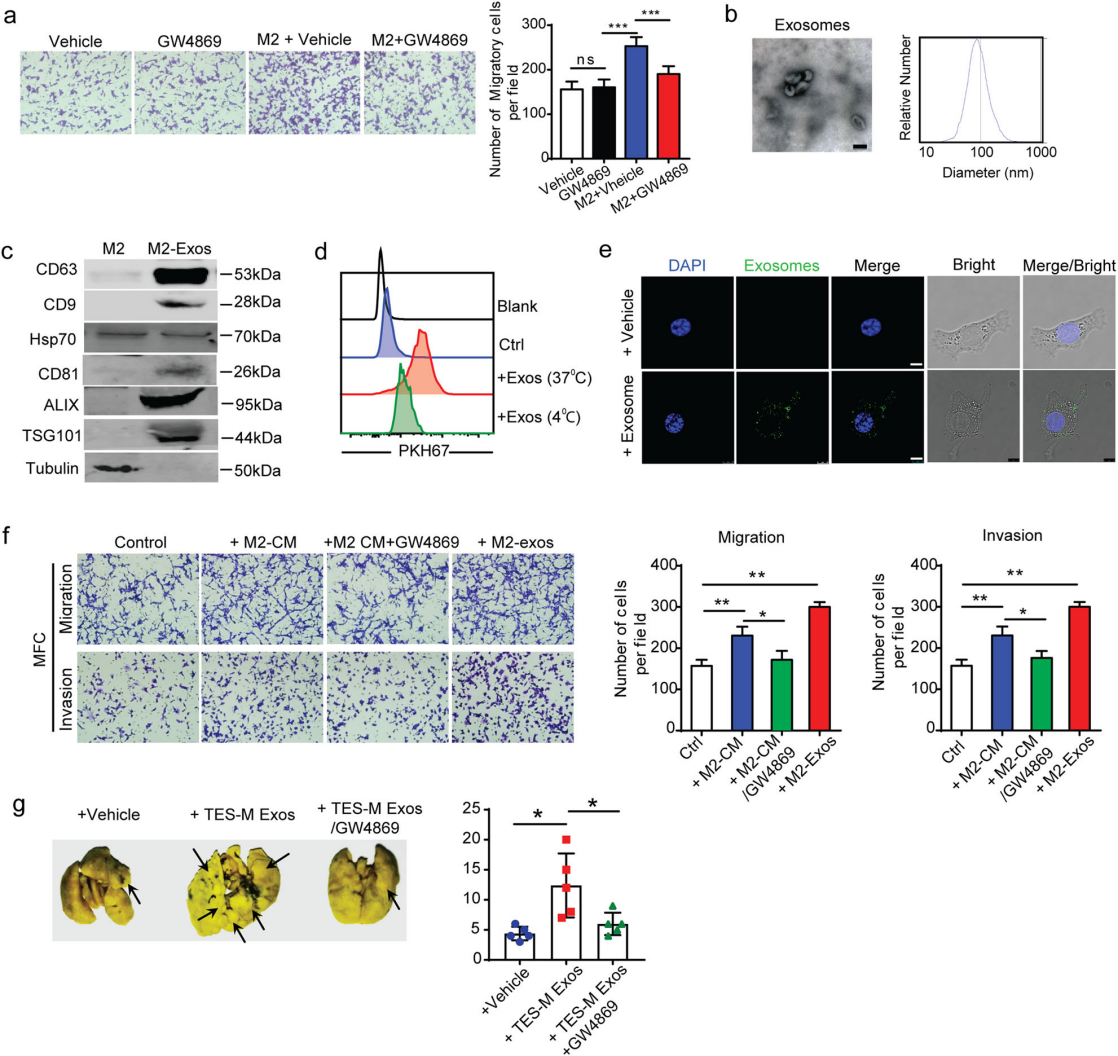
Exosomes are transferred from M2 macrophages to tumor cells and promote migration of GC cells. **a** Migration assay of MFC cells cocultured with M2 macrophages treated with or without 10 µM GW4869 (blocking exosome generation). Shown is the mean ± s.e.m. of three independent experiments. **b** Transmission electron microscopy image of exosomes. TRPS analysis of exosomes confirming the expected size range of 30–150 nm in diameter. **c** Western blot analysis of exosome markers in M2 macrophage-derived exosomes (M2-Exos) and cell lysates. **d** PKH67-labeled M2-Exos incorporation by MFC cells detected by flow cytometry. **e** Immunofluorescence images of exosome (green) uptake by MFC cells after treatment with PKH67-labeled M2-Exos. Scale bar represents 10 μm. **f** Migration and invasion assay of MFC cells pretreated with M2 macrophage-derived conditional medium or exosomes with or without GW4869 treatment, accompanied by quantification of migratory and invasive cells. Shown is the mean ± s.d. of two independent experiments. **g** Representative lung tissues and quantification of lung metastasis of mice administered MFC cells pretreated with or without TES treated macrophages-exosomes (TES-M Exos) or GW4869-treated TES-M Exos. (*n* = 5 in each group). Black arrows show the metastasis nodules in the lung. All values are depicted as the mean ± s.d. *P < 0.05, **P < 0.01, ***P < 0.001; by unpaired two-sided Student’s *t*-test (**g**) or one-way ANOVA with Tukey’s method for multiple comparisons (**a**, **f**)

To dissect the mechanism by which M2 macrophages promote migration of GC cells, we evaluated the soluble factors of M2 macrophages and did not find a significant impact. Emerging evidence suggests that exosomes have a central role in cell–cell communication in the TME^17,18^. To explore whether exosomes have a critical role in this effect, we blocked exosome formation by treating M2 macrophages with GW4869 (Supplementary Figure S3a). Following GW4869 treatment, M2 macrophages failed to promote the migration of GC cells (Fig. 3a). The purified exosomes from the conditioned medium of M2 macrophages displayed the typical morphology and size of exosomes (Fig. 3b) and contained CD63, CD9, CD81, TSG101, and ALIX, all of which are marks of exosomes (Fig. 3c, Supplementary Figure S3b).

To examine whether polarized M2 macrophages-derived exosomes (M2-Exos) can be taken up by GC cell, we pre-labeled M2-Exos with PKH67. As indicated by a shift in the peaks, M2-Exos was indeed taken up by MFC GC cell (Fig. 3d). Examination using fluorescence microscopy also confirmed the uptake of exosomes by MFC cells (Fig. 3e) or MGC cells (Supplementary Figure S3d). To determine whether exosomes produced by M2 macrophages are sufficient to induce GC cell motility, we treated MFC cells with isolated M2-Exos in vitro. Indeed, M2-Exos significantly increased the migration and invasion of MFC cells (Fig. 3f, Supplementary Figure S3c, e), whereas proliferation was not significantly affected (Supplementary Figure S3f, g). In addition, the similar pro-migratory potential was observed in MFC cell treated with exosomes derived from macrophages induced by tumor explant supernatant (Supplementary Figure S3c). However, GW4869 treatment blocked the pro-migratory effect of M2 macrophages-exosomes on GC cells (Fig. 3f). To demonstrate the effect of M2-Exos in vivo, we observed lung metastasis in mice after administration of MFC cells pretreated by M2-Exos with or without GW4869. As shown in Fig. 3g, increased lung colonization was found in mice inoculated with MFC cells treated with exosomes derived from macrophages induced by tumor explant supernatant (TES-M Exos), compared to untreated cells and GW4869-treated TES-M Exos. Collectively, these findings demonstrated that exosomes from M2 macrophages enhanced the aggressiveness of GC cells, highlighting a new mode of communication between macrophages and GC cells.

### Apolipoprotein E is enriched in M2 macrophages-derived exosomes and highly expressed in M2-polarized macrophage

**Fig. 4:**
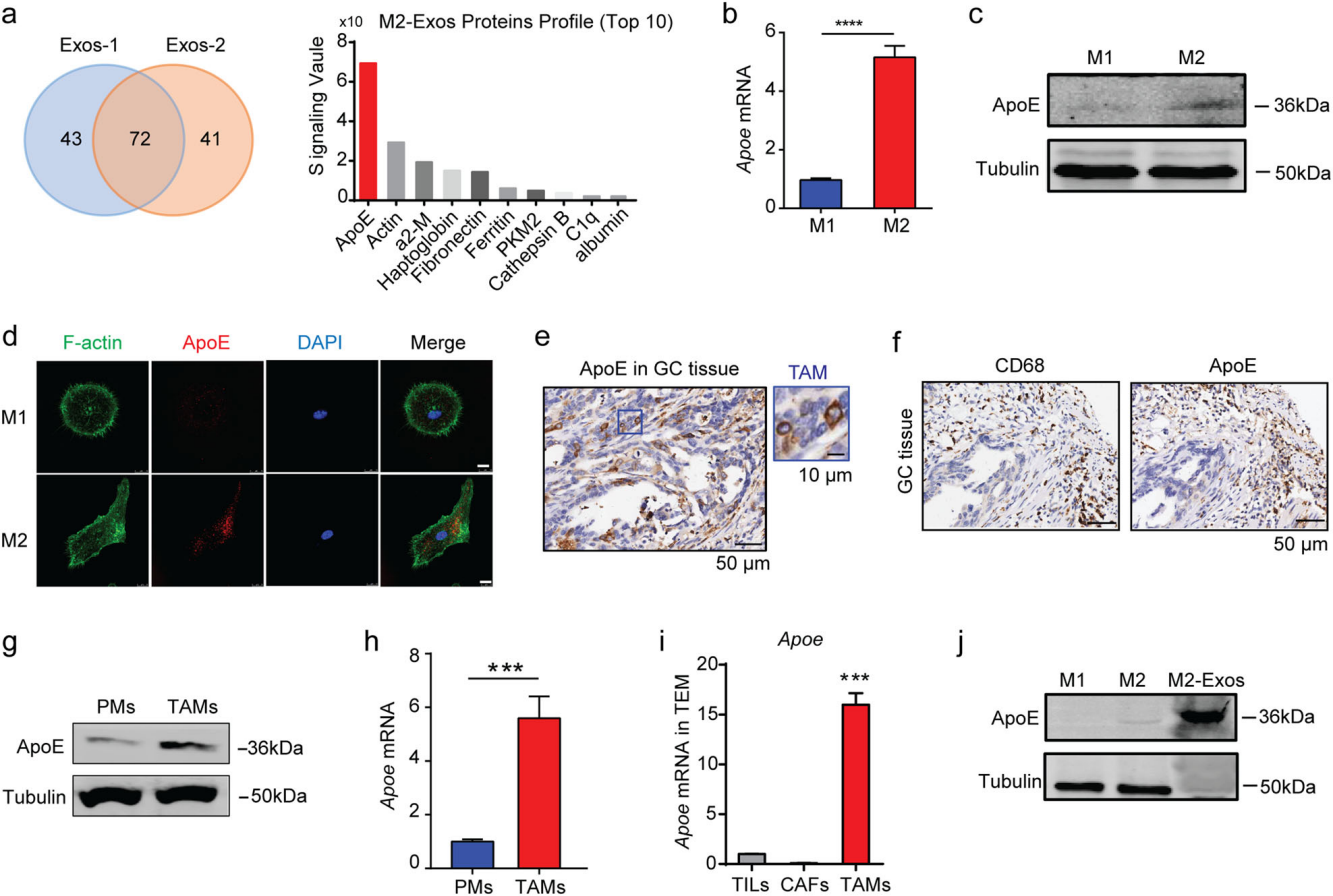
**Apolipoprotein E is enriched in M2 macrophages-derived exosomes and highly expressed in M2-polarized macrophage.** **a** The protein content of M2-Exos obtained from two isolation methods (Exo-1: ultracentrifugation and Exo-2: ExoQuick™) was determined by mass spectrometry. The top 10 most abundant proteins among a total of 73 common proteins are listed. **b**, **c** mRNA and protein expression of ApoE in M1 and M2-polarized macrophages. **d** Immunofluorescence of ApoE (red) in the M1 and M2 macrophages. Scale bars represent 10 μm. **e** IHC staining of ApoE for TAMs (blue square) and tumor cells (red square) from human GC tissues. Scale bars represent 50 μm (inset, 10 μm). **f** Representative IHC staining of CD68 and ApoE in serial sections of human GC tissues. **g**, **h** Western blot analysis (**g**) and quantification of gene expression (**h**) of ApoE in mouse peritoneal macrophage (PMs) and TAMs isolated from MFC tumor tissue. Shown is the mean ± s.e.m. of two independent experiments. **i** Quantification of gene expression of *APOE* in tumor-infiltrating lymphocytes (TILs), cancer-associated fibroblasts (CAFs), and TAMs isolated from human GC tissues. **j** Western blot analysis of whole-cell lysates of M1 or M2 macrophages and M2-Exos. Error bars represent mean ± s.e.m. **P < 0.01, ***P < 0.001, ****P < 0.0001; n.s. not significant; by one-way ANOVA with Dunnett’s multiple-comparison test (**i**) or Student’s *t*-test (**b**, **h**)

To unravel the molecular mechanism responsible for the pro-migratory effect of M2-Exos, we analyzed their protein content by mass spectrometry. Among the proteins identified, 72 overlapping proteins were identified and confirmed by two types of exosome purification methods (ultracentrifugation and ExoQuick™ Extraction) (Fig. 4a, Supplementary Table S4). Remarkably, we identified Apolipoprotein E (ApoE) at the highest level in M2-Exos (Fig. 4a). Interestingly, ApoE was typically predominantly expressed in M2 macrophages at the mRNA and protein level (Fig. 4b–d). Specifically, human GC tissues also revealed that ApoE was primarily expressed in TAMs (Fig. 4e, f). In line with this finding, a higher ApoE expression at the mRNA and protein level was also observed in TAMs from in vivo mouse GC model (Fig. 4g, h). Moreover, TAMs are the majority cell population expressing ApoE in the TME (Fig. 4i). Western blot also confirmed the predominant enrichment of ApoE in M2-Exos (Fig. 4j), however, soluble ApoE was undetectable in the conditional medium of M2 macrophage.

### Exosomal transfer of Apolipoprotein E from M2 macrophages promotes the migration of GC cells

**Fig. 5:**
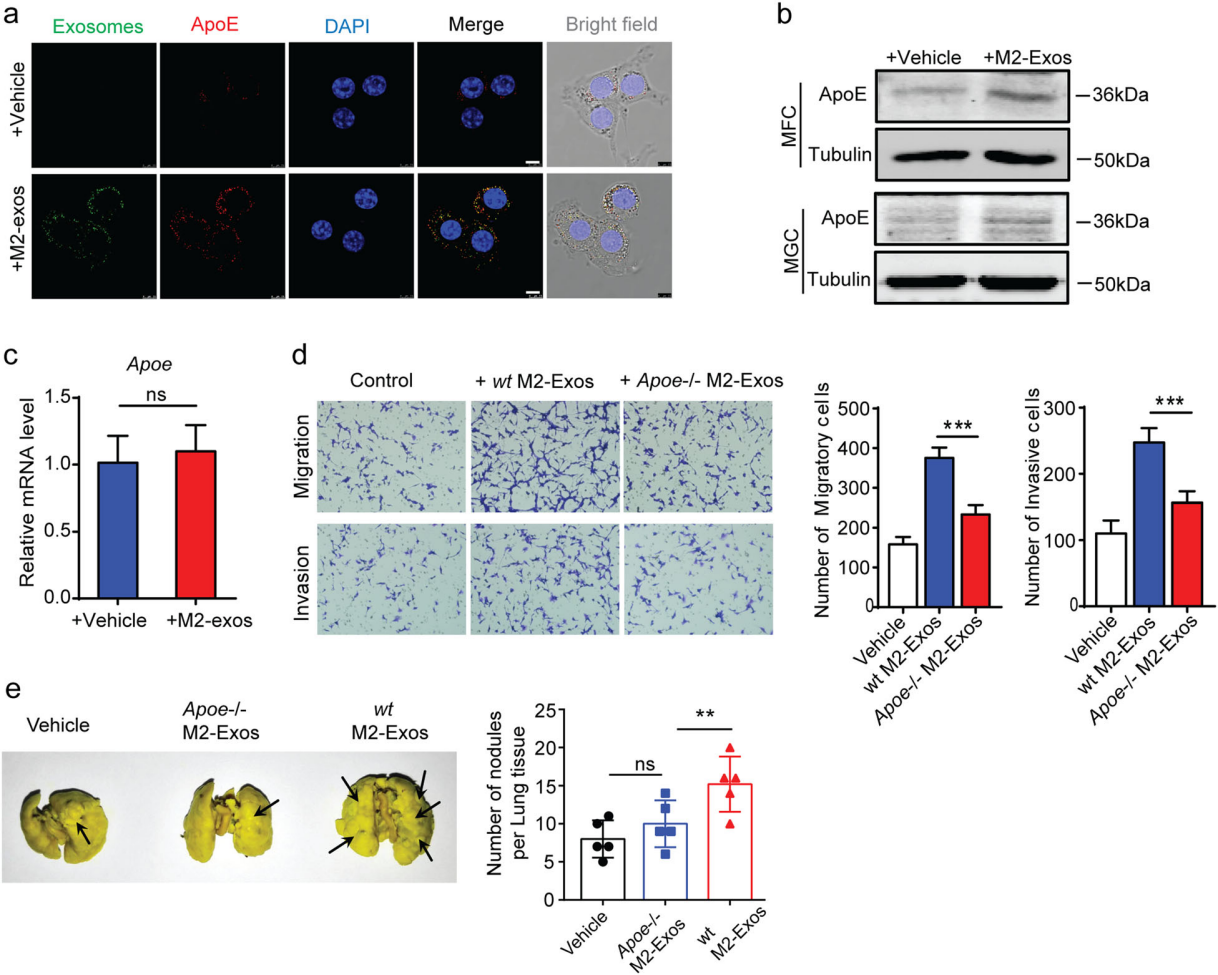
**Exosomal transfer of Apolipoprotein E from M2 macrophages promotes migration of GC cells.** **a** Immunofluorescence staining of ApoE in MFC cells cultured with M2-Exos (green) or vehicle. Scale bars represent 15 μm. **b** Western blot analysis of ApoE in MFC and MGC cells cultured with M2-Exos or vehicle control. **c** Real-time PCR analysis of ApoE mRNA in MFC cells cultured with M2-Exos or vehicle control. **d** Migration and invasion assay of MFC cells cultured with M2-Exos from *Apoe*^*−/−*^ or WT mice. Shown is the mean ± s.e.m. of three independent experiments. **e** Representative lung tissue images and quantification of lung metastasis in mice administered MFC cells pretreated with exosomes from *Apoe*^*−/−*^ or WT BMDMs (*n* = 5). Error bars represent mean ± s.e.m. **P < 0.01, ***P < 0.001, ****P < 0.0001; n.s., not significant; by one-way ANOVA with Dunnett’s multiple-comparison test (**d**, **e**) or Student’s *t*-test (**c**)

We hypothesized that M2-Exos might induce the increased migration of recipient GC cells through the transfer of functional ApoE. Colocalization of ApoE and exosomes was detected in MFC cells cocultured with M2-Exos (Fig. 5a), confirming that ApoE was transferred from M2 macrophages to GC cells via exosomes. In favor of this hypothesis, mouse or human GC cells treated with M2-Exos presented increased levels of ApoE protein (Fig. 5b), with no difference in the corresponding mRNA levels (Fig. 5c). Moreover, ApoE level was increased in MFC cells incubated with M2-Exos, accompanied by increased migration and invasion. To better evaluate the impact of ApoE on M2-Exos-mediated cell migration, *Apoe*^*−/−*^ macrophages derived from the BM of *Apoe*^*−/−*^ mouse were used and polarized. Although ApoE knockout did not affect the polarization of macrophages (Supplementary Figure S4a–c) and *Apoe*^*−/−*^ M2-Exos did not affect proliferation or apoptosis (Supplementary Figure S4d–f), we did not observe the pro-migratory effect of *Apoe*^*−/−*^ M2-Exos on recipient GC cells (Fig. 5d), demonstrating the critical role of ApoE in this process. We wondered whether recombinant ApoE was sufficient to stimulate and recapitulate the migratory phenotype of GC cells. To test this possibility, we performed migration assay to show that there were no significant differences on the migration of GC cells with or without treatment of different concentration of recombinant ApoE (Supplementary Figure S5a, b), meaning recombinant ApoE is not sufficient to stimulate the migratory phenotype of GC. *Apoe*^*−/−*^ M2-Exos induced a significant reduction in lung metastasis compared to WT M2-Exos in vivo (Fig. 5e), further supporting the link between M2-exosomal ApoE and migration of GC cells.

### The PI3K-Akt signaling pathway mediates macrophage exosomal ApoE-induced pro-migratory of GC cells

**Fig. 6:**
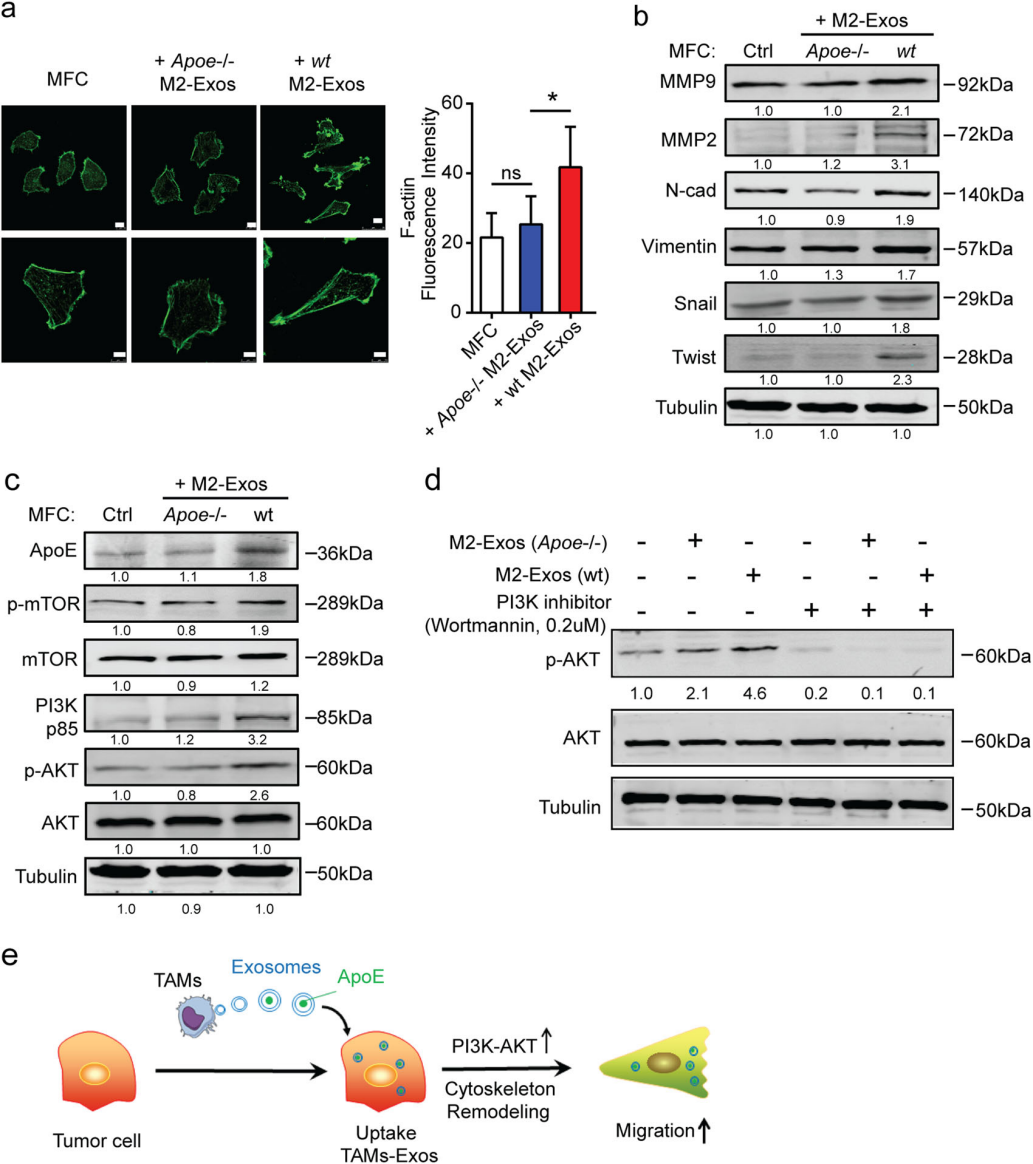
**The PI3K-Akt signaling pathway mediates macrophage exosomal ApoE-induced aggressiveness in GC cells.** **a** Representative immunofluorescence and quantification of actin staining (green) using phalloidin (scale bar, 10 µm) in MFC cells treated with M2-Exos from *Apoe*^*−/−*^ or WT BMDMs. Shown is the mean ± s.d. of three independent experiments. **b** Western blot analysis of EMT proteins after MFC cells were treated with M2-Exos from *Apoe*^*−/−*^ or WT BMDMs. **c** Western blot analysis of PI3K-Akt signaling proteins after MFC cells were treated with exosomes derived from *Apoe*^*−/−*^ or WT M2 macrophages. **d** Western blot analysis of pAkt/Akt proteins in MFC cells treated with 0.2 µM of PI3K inhibitor (wortmannin) with or without M2-Exos from *WT* or *Apoe*^*−/−*^ M2 macrophage. **e** Proposed working model. Exosomes secreted by M2-type TAMs transfer ApoE into adjacent GC cells, leading to PI3K-Akt-mTOR signaling pathway activation in tumor cells. PI3K-Akt pathway activation in tumor cells increases cytoskeletal remodeling, facilitating the migratory potential of the tumor cell. Error bars represent mean ± s.d. *P < 0.05, **P < 0.01; n.s. not significant; by one-way ANOVA with Dunnett’s multiple-comparison test (**a**)

We further sought to identify the mediator of the M2-exosomal ApoE-driven pro-migratory potential. Our previous studies and other recent research have suggested that remodeling of the actin cytoskeleton is involved in the metastasis of cancer cells^19–21^. We found that WT M2-Exos-treated GC cells had markedly increased the intensity of actin compared with *Apoe*^*−/−*^ M2-Exos-treated GC cells (Fig. 6a). In addition, we showed that multiple proteins associated with epithelial–mesenchymal transition (EMT) were upregulated in GC cells treated with M2-Exos (Fig. 6b). However, *Apoe*^*−/−*^ M2-Exos have no significant effect on cytoskeletal remodeling and EMT proteins (Fig. 6a, b). In addition, we evaluated the cytoskeletal remodeling-related pathway based on previous findings. We did not find a significant difference in the ATF3-GSN pathway (Supplementary Figure S6a), which has been shown to be involved in EMT-mediated cytoskeletal remodeling^19^. Growing evidence support activation of the PI3K/Akt pathway as having a key role in the modulation of cytoskeletal rearrangement^22^. We found that M2-Exos-treated GC cells exhibited increased phosphorylation of the PI3K-Akt signaling pathway proteins compared to vehicle-treated GC cells (Fig. 6c). However, neither *Apoe*^*−/−*^ M2-Exos (Fig. 6c) nor recombinant ApoE (Supplementary Figure S6b) had a significant effect on the PI3K-Akt signaling pathway, indicating that M2-Exos-derived ApoE activated the PI3K-Akt signaling pathway in recipient GC cells.

The preceding data raised a question concerning whether upregulation of ApoE was sufficient to confer pro-migratory traits to GC cells. We confirmed that forced expression of ApoE endowed MFC cells with increased migration potential (Supplementary Figure S6c, e), accompanied by enhanced cytoskeletal rearrangement (Supplementary Figure S6d, f). We also found that PI3K inhibitor treatment reverses the activation effect of M2-Exos on PI3K-Akt signaling pathway in GC cell (Fig. 6d). In sum, we show that exosomes secreted by M2-type TAMs transfer functional ApoE into GC cells, leading to PI3K-Akt signaling pathway activation, and facilitating migration of GC cells (Fig. 6e).

## Discussion

We demonstrate that interaction between TAMs and GC cells by exosomal ApoE results in PI3K/Akt signaling events that drive TAMs-mediated migration. First, tumor-infiltrating M2 macrophages express significantly higher levels of ApoE and transfer functional ApoE exosomes to neighboring GC cells. Second, M2-exosomal transfer of ApoE triggers PI3K-Akt signaling activation, which facilitates cytoskeletal remodeling, resulting in increased migration potential of GC cells. Our findings provide a biochemical explanation for the clinical association between ApoE expression in GC tissue and a higher potential for invasion and metastasis^9^.

TAM-featured inflammation is known as the hallmark of cancer^22^. Although M1 macrophages are linked with tumoricidal activity^23^, M2-polarized macrophages have been shown to be associated with cancer progression and metastasis^24,25^. Interactions with stromal cells are critical for the development of metastasis in the TME^2,26^. Increasing evidence has revealed an important role for stromal cell exosomes as mediators of cell–cell communication within TME^27–29^. Here, we show the importance of M2 macrophage-derived exosomes in malignant progression of GC. Exosomes are a key player in the dialog between macrophages and cancer cells in GC. A recent study has shown that exosomes shed by epithelial ovarian cancer cells induced the polarization of tumor-promoting M2 macrophages^15^, indicating a role for exosomes in both directions of the crosstalk between macrophages and cancer cells.

Functionally, exosomes can transfer a variety of proteins, DNA, and RNA, which have intriguing and elaborate roles in cancer progression. In this study, the protein that was significantly enriched in M2 macrophage-derived exosomes was ApoE. ApoE is a major protein component of very-low-density lipoproteins and high-density lipoproteins. The antiatherogenic activity of ApoE was shown to induce macrophage conversion from the proinflammatory M1 to the anti-inflammatory M2 phenotype^30^, which is consistent with the specific enrichment of ApoE in M2 macrophages in our findings. It is well established that ApoE is a polymorphic molecule that has critical roles in cardiovascular and neurodegenerative disorders^31^. The role of ApoE in cancer cells is still under debate. Numerous studies have shown that ApoE inhibits tumor growth^32,33^, while intriguingly, other evidence has indicated that ApoE was required for proliferation of cancer cells^34^. Therefore, this duality of ApoE activity on cancer cells is tissue-specific. In GC, ApoE has recently been identified as a potential tumor-associated marker. However, little is known about the effect of external ApoE on tumor cells. Macrophages are the main source of ApoE in the gastrointestinal tract^35^, indicating the important role of ApoE from macrophages in gastrointestinal cancer. Interestingly, we found that M2-exosome-mediated pro-migratory ability was dependent on ApoE, as confirmed in vivo and in vitro. Thus, our study identified a critical role of macrophage exosome-derived ApoE, but not endogenous ApoE in tumor cells, in mediating the crosstalk between TAMs and GC cells, promoting metastasis.

Exosomes can have an important role in cell signal transduction^36^. However, the role of exosomal transferable protein in driving the migratory signaling pathway is less well understood. Although ApoE is a key regulatory protein in lipoprotein metabolism^37,38^, nonlipid-related functions have also been attributed to ApoE^37^. ApoE affects several signaling cascades, including by increasing disabled phosphorylation and by activation of the ERK1/2 pathway^39^. Here, we demonstrated that TAM exosome-derived ApoE activated PI3K/AKT/mTOR signaling pathway of GC cells. Moreover, ApoE, transferred by TAM exosomes, promotes the migratory ability GC cells through cytoskeletal remodeling. PI3K/Akt/mTOR signaling has been confirmed as a critical regulator during tumor progression, including cell–cell adhesion, proliferation, and migration^40^. As such, PI3K signaling was determined to be associated with the metastatic cascade in gastric carcinoma, which includes proteolytic activity and cytoskeletal remodeling^41^. It is not yet clear whether other signaling pathways may also be involved in the ApoE-mediated promotion of GC cell migration. Thus far, we also do not know whether TAMs exosomal ApoE is directly transferred and uptake or mediated by ApoE receptor. However, we demonstrated that recombinant ApoE was not sufficient to stimulate the migratory phenotype of GC. The previous study has proven that exogenous ApoE and endogenous ApoE were confined in separate cellular compartments resulted in different function^42^. A more in-depth analysis is required to justify the mechanism of ApoE in M2 exosome-mediated migration. Clinically, on the basis of gene expression analysis, ApoE has been identified as a potential tumor-associated marker in GC^9,43^. In particular, ApoE was closely correlated with metastasis^44^. Here we clarified that ApoE was primarily expressed in TAMs and tumor cells adjacent to TAMs in GC.

In summary, we provide evidence that M2 macrophage-derived exosomes promote the migration of GC cells via the PI3K-Akt signaling pathway. We identify a critical role of ApoE from M2 exosomes in exerting the driving force that promotes migration via cytoskeletal rearrangement. Admittedly, other TAM-derived chemokines or growth factors may also contribute via other signaling pathways. Understanding the bi-directional communication between TAMs and tumor cells as well as the regulation of tumor cell metastasis may lead to more effective strategies for macrophage-based cancer therapy. This study provides the groundwork for macrophage exosomes research toward a further understanding of their clinical and pathological importance. Further studies are needed to evaluate whether targeting ApoE or inhibiting exosomes released by TAMs can be manipulated to inhibit cancer metastasis.

## Materials and methods

### Patient cohorts

In cohort-I, 87 gastric tumor tissues were obtained from patients with GC treated at Xinhua Hospital, Shanghai Jiao Tong University School of Medicine, China, between 2009 and 2014. In verified cohort II, GC tissue microarray chips containing 40 pairs of tumors and matched adjacent tissues were obtained from the Shanghai Outdo Biotech Company (Shanghai, China). The clinicopathological and follow-up data of patients were prospectively collected. All patients were diagnosed by pathological analyses based on the International Union against Cancer (UICC)-defined TNM criteria. Non-invasive GCs were confined to the mucosa and/or submucosa without lymph node metastases, irrespective of the tumor size. Invasive GC was defined by submucosal invasion, submucosa invasion, lymphatic and venous invasion. In this study, distant metastasis was defined that GC had spread to distant parts of the body in addition to the area around the stomach including liver, peritoneum, lung, and bone. Lymph node metastasis was identified that metastasis to intra-abdominal lymph nodes, including hepatoduodenal, retropancreatic, mesenteric, and para-aortic. The study protocol conformed to the ethical guidelines of the Declaration of Helsinki and was approved by the Institutional Review Board and Ethics Committee of Xinhua Hospital.

### Identification of immune cells enrichment

Then enrichment of immune cell subpopulations was analyzed in 19 solid tumors (Supplementary Table S1) by web-accessible relational database TCIA (https://tcia.at/), which provided the results of comprehensive immunogenomic analyses of next-generation sequencing data (NGS). In 142 STAD patients, macrophage and others immune cell types were identified by using single sample GSEA (ssGSEA) and the deconvolution method, expression of predefined immune subsets that are over-represented in the TME^16^.

### Cell culture and mouse strain

The mouse gastric carcinoma MFC cell and human GC cell line MGC-803 (MGC) were obtained from Cell Bank of Type Culture Collection of Chinese Academy of Sciences and tests for mycoplasma contamination were negative. Human MGC-803 cell line was validated using STR DNA fingerprinting. The cells were maintained in DMEM supplemented with 10% fetal bovine serum (FBS) and penicillin with streptomycin (Gibco). The plasmid of ApoE overexpression (ApoE OE) and respective control vectors were provided by Shanghai GeneChem Co., Ltd. (Shanghai, China). Lipofectamine 3000 (Life Technologies, Carlsbad, CA, USA) was used for plasmid transfection. Male 8-week-old C57BL/6 mice and *Apoe*^*−/−*^ mice with a C57BL/6 background were purchased from Vital River Laboratory Animal Technology Co. Ltd. (Beijing, China). Male 8-week-old 615 mice were purchased from Military Medical Sciences (Beijing, China). All animals’ experiments were carried out according to the Principles of Laboratory Animal Care (China) and approved by the Ethics Committee of Xinhua Hospital, Shanghai Jiao Tong University School of Medicine.

### Macrophage polarization assay

Murine bone marrow-derived macrophages (BMDMs) were prepared and plated in bone macrophage medium (BMM) consisting 50 ng/ml M-CSF. After 7 days in culture, cells were induced towards a polarized phenotype with the addition of 100 ng/ml LPS plus 20 ng/ml IFN-γ (for M1 polarization) or 20 ng/ml IL-4 plus 20 ng/ml IL-13 (for M2 polarization). Human polarized macrophages were prepared from peripheral blood mononuclear cells (PBMCs) of healthy donors. The polarization of the resulting monocyte-derived macrophages was achieved as described^45^. In some experiments, BMDMs was cultured with tumor explant supernatants (TESs) or tumor-conditioned media (TCM). Tumor explants were prepared from freshly isolated subcutaneous MFC tumors. MFC tumor explants were removed and digested, and then tumor samples were pressed through a 70um nylon filter (BD Biosciences) to create a single cell suspension. Cells were cultured in RPMI 1640 with 10% FBS and 1% penicillin plus streptomycin overnight. The cell-free supernatant was collected to prepare tumor explant supernatant. MFC cells were grown in DMEM-complete medium. After one day, the medium was recovered and filtered through a sterile 0.22 μm syringe filter to prepare tumor-conditioned medium (TCM).

### Exosome preparation and analysis

Exosomes were collected by density gradient ultracentrifugation according to previously published protocol^46^. In brief, the polarized macrophages were incubated for 48 h in complete PRMI1640 medium with 10% FBS that was previously depleted of contaminating vesicles by overnight centrifugation at 100,000×*g*. The conditioned medium was collected and centrifuged at 800×*g* for 10 min, followed by a centrifugation step of 3000×*g* for 30 min to remove cell debris. Next, the supernatant was filtered using a 0.22-µm filter (Millipore). The exosomes were pelleted by ultracentrifugation at 100,000×*g* for 90 min, washed in PBS, pelleted again and re-suspended in PBS. Measurement of the exosome particle number was performed using a CD63 ExoELISA Complete Kit (System Biosciences, USA) following the manufacturers’ instructions. For Nano-LC–MS/MS analysis, exosome pellets were also isolated using ExoQuick-TC TM (System Bioscience) according to the manufacturer’s protocol. For exosome uptake experiments, exosome preparations were labeled with PKH67 Fluorescent Cell Linker Kits (Sigma-Aldrich) according to the manufacturer’s instructions, followed by washing through Exosome Spin Columns (MW3000) (Invitrogen, USA) to remove excess dye. Next, exosomes were incubated with GC cells, which were examined under a confocal microscope or analyzed using flow cytometry at the indicated time points.

### Transmission electron microscopy (TEM)

For TEM, 10 μl of exosome suspension was adsorbed onto carbon-coated copper grids (200 mesh) for 1 min. Samples were washed with double-distilled water and negatively stained with 2% uranyl acetate solution for 1 min. Grids were visualized at ×87000 in a Phillips Tecnai transmission electron microscope at 80 kV. Tunable resistive pulse sensing (TRPS) was used and analyzed the concentration and size distribution of particles by an NP100 nanopore (qNano, Izon Science Ltd) at a 45 mm stretch.

### Nano-LC–MS/MS analysis

Fifty micrograms of proteins from exosomes were submitted for proteomic analysis using Nano-LC-MS/MS. Experiments were performed on a Q Exactive mass spectrometer that was coupled to Easy nLC (Thermo Fisher Scientific) in Shanghai Applied Protein Technology Co., Ltd. MS/MS spectra were searched using MASCOT engine (Matrix Science, London, UK; version 2.2) against the UniProt mouse sequence database (81,144 total entries downloaded 05/23/2016). For protein identification, the following options were used. Peptide mass tolerance = 20 ppm, MS/MS tolerance = 0.1 Da, enzyme = trypsin, missed cleavage = 2, fixed modification: carbamidomethyl (C), variable modification: oxidation (M). The protein identification criteria that we used were based on score ≥20. Protein identification results were extracted from the mascot data file with in-house software (Build Summary).

### Cell migration and invasion assay

Cell migration and invasion assays were conducted on 24-well Transwell cell culture chambers with 8-μm sized pores with or without precoated Matrigel (Corning, USA). GC cells were trypsinized and washed three times with PBS, and then 5 × 10^4^ cells were suspended in 500 μl of medium and added to the upper inserts; M1 or M2 macrophages were added to the lower inserts. For the control, 750 µl of medium with 10% FBS was placed in the lower chamber. In addition, GC cells were harvested after 24 h of coculture with supernatant or exosomes of M2 macrophages, suspended in 500 μl of FBS-free medium and added to the upper inserts, and 750 µl of medium with 10% FBS was placed in the lower chamber. After 24 h of incubation, the cells remaining in the upper chamber were removed, and the cells on the lower surface of the chamber were fixed with 4% paraformaldehyde and stained with 0.5% crystal violet. At least five random microscopic fields (magnification ×200) were photographed, and the cells were counted. Three independent experiments were performed. For inhibition of exosome generation, macrophages were treated with culture media containing 10 μM GW4869 (Sigma).

### RNA extraction and quantitative real-time PCR

RT-PCR analyses were performed as previously described^47^. Primers sequences of mentioned genes are described in Supplementary Table S2.

### Western blot

Briefly, equal amounts of cells or exosomes were harvested in standard RIPA buffer supplemented with protease and phosphatase inhibitor cocktails (Roche). WB analyses were performed as previously described^19^. The quantification of each protein band was performed using ImageJ software (USA). All antibodies used for western blot are listed in Supplementary Table S3.

### Immunofluorescence assay

Specimens were prepared as previously described^20^. F-actin was visualized by staining with Alexa 488 phalloidin (Thermo Fisher, USA) according to the manufacturer’s guidelines. Images were captured using a Leica SP5 Laser scanning confocal microscope. Actin filaments were quantified after staining with phalloidin using ImageJ software as previously described^20^.

### Isolation of tumor-infiltrating macrophage cells

Mouse or human fresh tumor samples were minced with scissors before incubation with 1.67 U/ml Liberase (Roche) and 0.2 mg/ml DNase (Roche) in RPMI for 30 min at 37 °C. Tumor samples were filtered through a 70 μm nylon filter (BD Biosciences). After red blood cell lysis, all samples were washed and re-suspended in FACS staining buffer for flow cytometry or real-time PCR. Peritoneal macrophages were collected 96 h after i.p. injection of a 3% thioglycollate solution. Cells were collected from the peritoneal cavity in 10 ml of PBS and macrophage enrichment was performed by plating cells in RPMI with 10% FBS and penicillin/streptomycin. After 2 h, non-adherent cells were removed with three PBS washes, and cells were analyzed by flow cytometry and RT-PCR.

### Flow cytometry staining and analysis

Specimens were prepared as previously described^48^. Labeled cells were analyzed on a BD FACSCanto II Flow Cytometer using BD FACSDiva software (BD Biosciences), and the data were processed using FlowJo software (Treestar). All antibodies used for flow cytometry are listed in Supplementary Table S3.

### Immunohistochemical staining

Specimens were prepared as previously described^20^. Automated image acquisition was performed using an Aperio ScanScope XT Slide Scanner system with a ×20 objective (Aperio Technologies).

### Tumor challenge and treatment experiments

To examine the metastatic ability of the GC cells, male 8-week-old 615 mice intravenously injected via the tail vein with 3 × 10^5^ MFC cells treated with or without indicated exosomes. All mice were killed at 6 weeks, and the lungs were excised. Then, 5–10 mouse lungs from each group were grossly examined for metastatic lesions, and the number of visible metastatic nodules were counted. For peritoneal metastasis assays, 3 × 10^5^ MFC treated with or without exosomes in 0.2 ml of PBS were injected into the peritoneal cavity of 8-week-old 615 mice. The mice were killed four weeks later, and the amount of ascites and the number of visible metastatic nodules were recorded. All experiments involved 5–10 mice per group.

### Statistical analyses

All statistical analyses were run using GraphPad Prism 7.0 software and displayed as the mean and s.e.m. The statistical significance of the difference was assessed using Student *t*-test, and the one-way ANOVA with Tukey post-test was conducted for multiple comparisons. For the survival analysis, Kaplan–Meier survival curves were calculated, and significance was determined by log-rank test. A significant difference was considered when the p-value was less than 0.05 and was represented by *P < 0.05, **P < 0.01, ***P < 0.001, and ****P < 0.0001.

## Electronic supplementary material

Supplemental information(DOC 3324 kb)
