# Supplementary material for: Tumor-associated macrophages-derived exosomes promote the migration of gastric cancer cells by transfer of functional Apolipoprotein E
Source: Cell Death Dis. 2018 Mar 22;9(4):434. doi: 10.1038/s41419-018-0465-5 (PMC5864742; doi:10.1038/s41419-018-0465-5)
Supplement: Supplementary file 1 — Supplemental information(DOC 3324 kb) [file 41419_2018_465_MOESM1_ESM.doc]

**Supplementary Information for: Peiming Zheng *et al*.**

**Supplementary Figures.**

**Figure S1.**

**Figure S2.**

**Figure S3.**

**Figure S4.**

**Figure S5.**

**Figure S6.**

**Supplementary Tables.**

**Table S1.**

**Table S2.**

**Table S3.**

**Table S4.**

**Supplementary Figures**

**
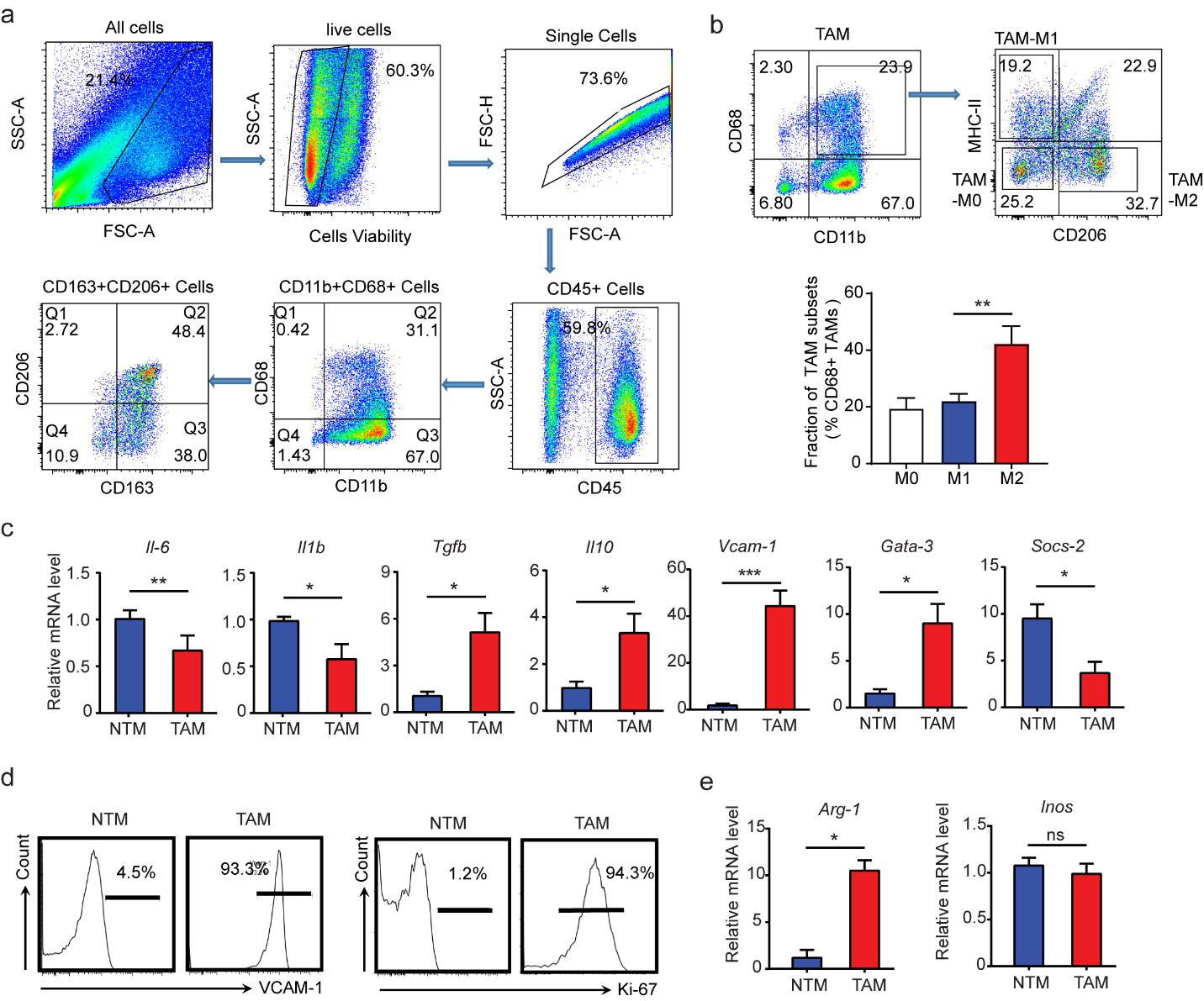
**

**Figure S1: The characterization of M2 type tumor-associated macrophages**

(a) The flow cytometry gating strategies for analyzing the markers of tumor associated macrophages (TAMs). Initial gating was on immune cells as approximated by scatter, then dead cells and doublets were eliminated. Next, CD45+ cells were selected, CD11b+CD68+ double positive cells were gated, and then analyzed the CD206+CD163+ as tumor associated macrophages. (b) Representative flow cytometry analysis and quantification of CD206, MHC class II expression in CD11b+CD68+ macrophages. (c) Quantification of gene expression of *socs-2, Il-6, Il-1b* (gene for M1 polarized macrophage) or Tgfb, Il10, *vcam-1, gata-3* (gene for M2 polarized macrophage) in normal tissue resident macrophages (NTM) and tumor-associated macrophages (TAM) isolated from human gastric cancer tissues. Data were relative to Gapdh expression and normalized versus the mean of monocytes. (d) Flow cytometry analyses of VCAM-1 (marker for M2 polarized macrophage), Ki-67 (proliferation marker) in NTM and TAM. (e) Quantification of gene expression of *arg-1* (gene for M2 polarized macrophage) or *inos* (gene for M1 polarized macrophage) in NTM and TAM isolated from mouse gastric cancer tissues (MFC cell xenograft model). Error bars represent mean ± s.d.; **P* < 0.05; ****P* < 0.001; n.s., not significant; by unpaired two-sided Student’s t-test (c, e).

**
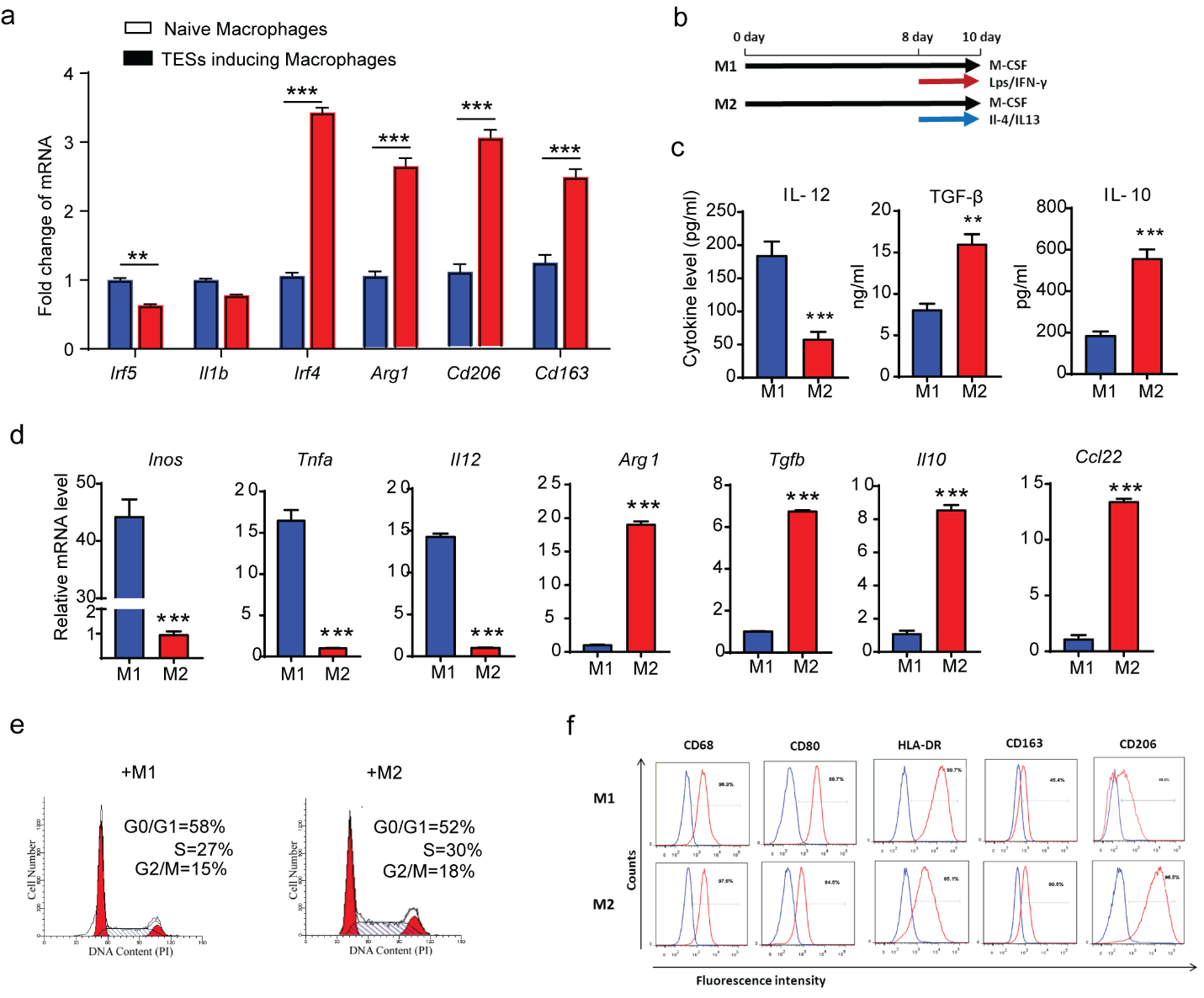
**

**Figure S2. Macrophage polarization was set up in vitro and exhibited a different effect on the gastric cancer cell.**

(a) Gastric cancer explant supernatant could induce the generation of M2 polarized macrophage. mRNA expression of the indicated typical M1 and M2 markers in macrophage cultured for 48 h with 30% tumor explant supernatant (TESs) from MFC tumor mice. Samples were evaluated in triplicate by quantitative RT-PCR (qRT-PCR), and the results were normalized to the level of expression of β-actin. (b) M-CSF-cultured macrophages from murine bone marrow or human peripheral blood monocytes (PBMCs) were stimulated with LPS and IFN-γ to differentiate to M1 polarized macrophage (M1), and stimulated by IL-4 and IL-13 to differentiate to M2 polarized macrophage (M2). (c) Cytokine analysis of M1 and M2 polarized macrophages from mouse BM by ELISA. (d) Gene expression profile of *inos, tnf-α, il-12* (gene for M1 polarized macrophage) or *arg-1, tgf-beta, il-10, ccl-22* (gene for M2 polarized macrophage) in M1 and M2 polarized macrophages from mouse BM. 18sRNA was assayed as a control. (e) Cell proliferation of MFC cells co-cultured with M1 or M2 macrophages was analyzed by flow cytometry. (f) FACS analyses of makers of M1- or M2-polarized macrophages from human peripheral blood monocytes. Error bars represent mean ± s.d.; **P* < 0.05, ***P* < 0.01, ****P* < 0.001; by unpaired two-sided Student’s t-test (a, c, d).

**
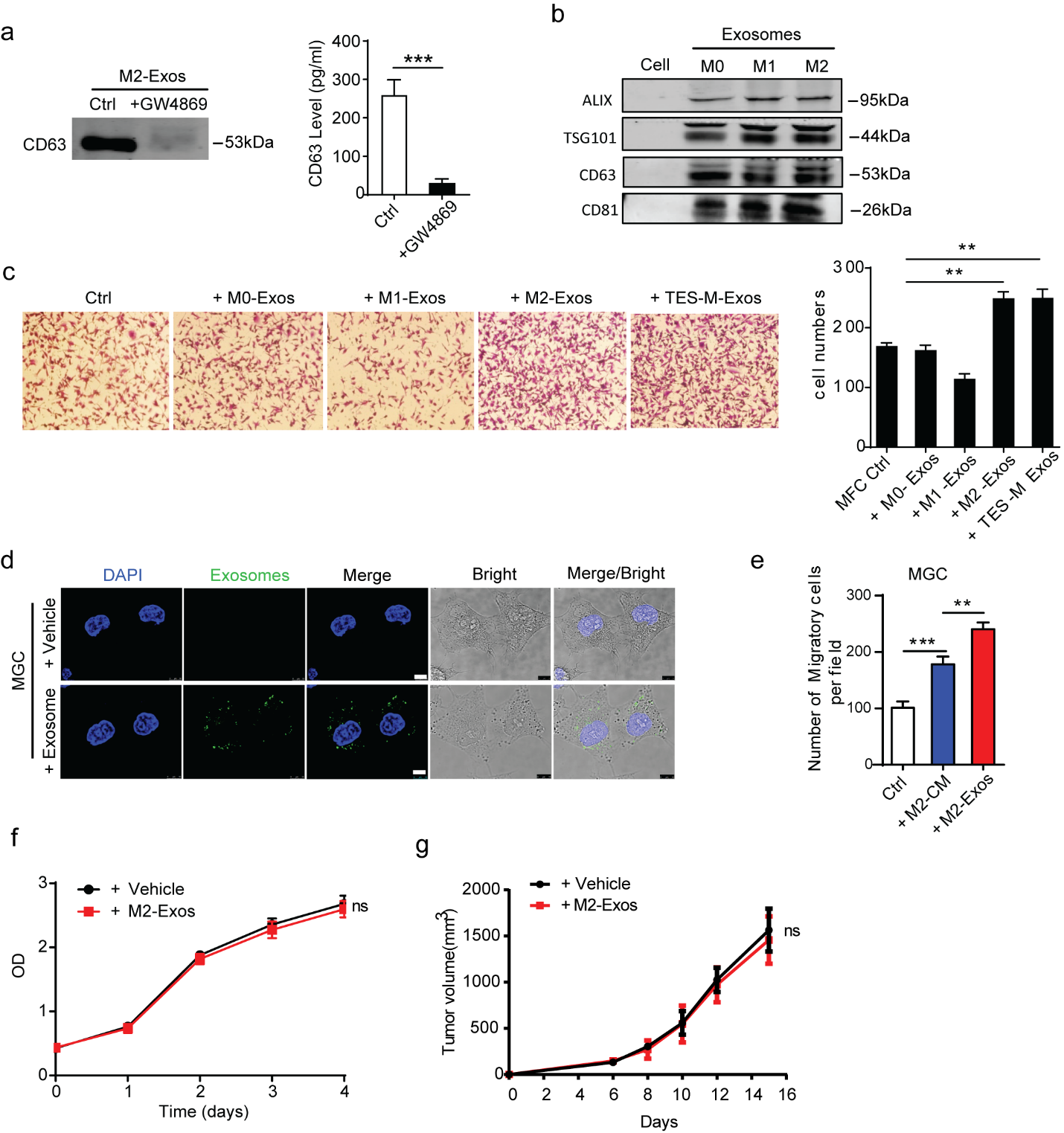
**

**Figure S3. Exosomes from M2 macrophages uptake by tumor cell enhanced the migratory ability without the effect on tumor cell proliferation in vitro or tumor growth in vivo.**

(a) Western blot analysis of CD63 expression in M2 macrophage-derived exosomes (M2-exos) pretreated with or without GW4869, accompanied by the quantification of exosomal marker CD63 as analyzed by ExoELISA CD63 kit. (b) Western blot analysis of exosome markers in M0, M1, M2 macrophage-derived exosomes (M2-exos) and cell lysates of naive macrophages. (c) Migration assay of MFC cells treated with M0-exosome, M1-exosome, M2-exosomes, or tumor explants supernatants induced macrophage-derived exosome (TES-M Exo), accompanied by quantification of migratory MFC cells with indicated treatment. Shown is the mean ± SEM of three independent experiments. (d) Representative immunofluorescence imaging for exosome (green) uptake by MGC cells with the treatment of PKH67-labeled M2 polarized macrophage-exosome. (e) Migration assay of MGC cells with M2 macrophages derived exosome (M2-exos) from human PBMC, with quantification of migratory cells. (f) The proliferation of MFC cells with or without the treatment of M2 macrophage-derived exosomes was detected by MTT assay. (g) Measurement of tumor growth in mice inoculated with MFC cells that were treated with vehicle or M2 macrophage-derived exosome (M2-exos).

**
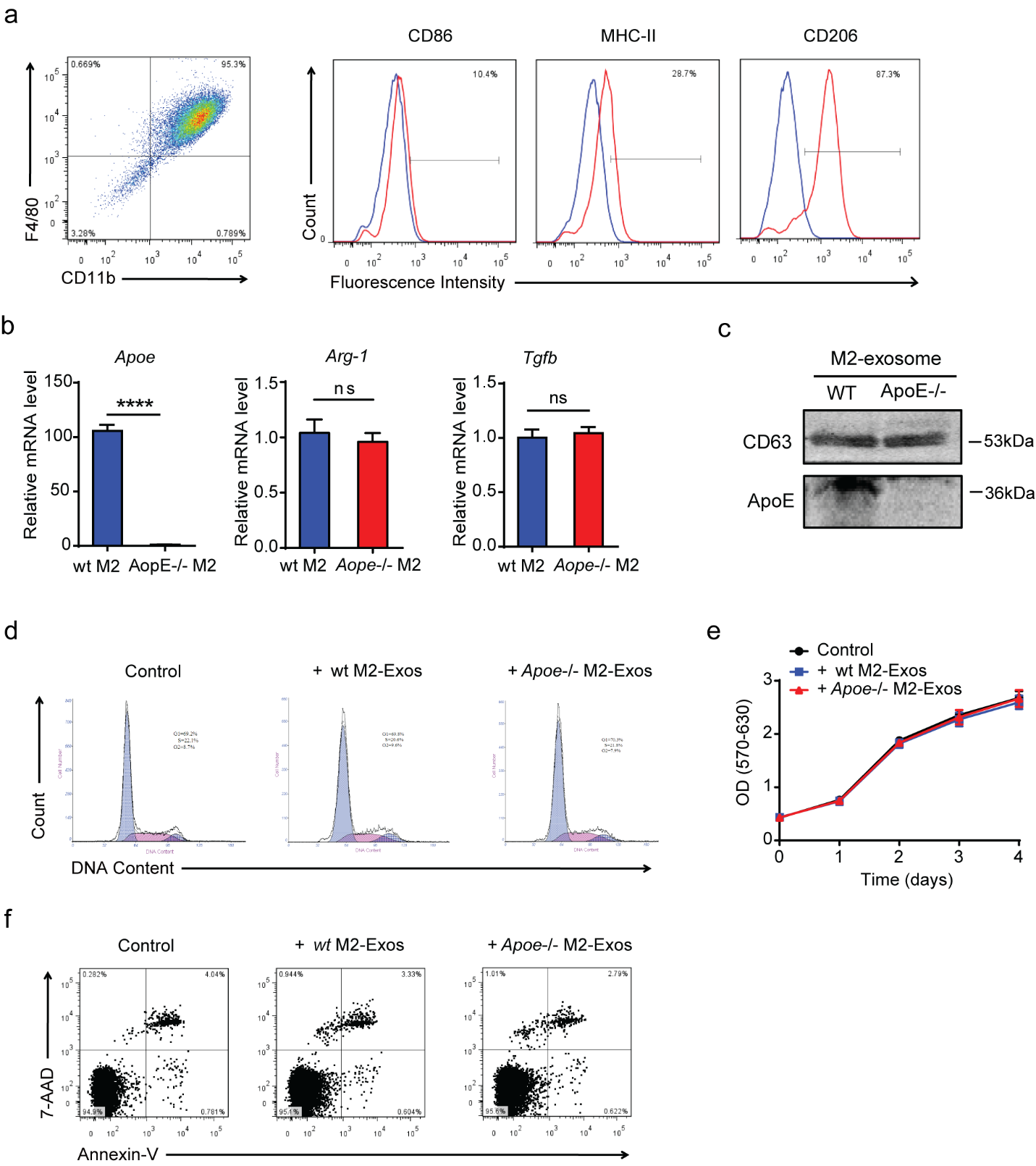
**

**Figure S4. Exosome from M2 polarized macrophage of ApoE^-/-^ mouse, which exhibits the normal phenotype of macrophage, did not effect on tumor cell proliferation in vitro.**

(a) FACS analyses of CD86, HLR-DR, and CD206 in M2 polarized macrophage from ApoE^-/-^ mouse BM with Th2 cytokines treatment. (b) Relative mRNA expression of ApoE, Arg-1, Tgf-beta in polarized macrophage derived from BM of wt or ApoE-/- mice. (c). Western blot analysis of CD63 and ApoE expression in polarized macrophage derived from BM of wt or ApoE-/- mice. (d). Representative cell cycle analysis of MFC cell with the treatment of M2-Exosome from wt or ApoE^-/-^ mouse. (e) The proliferation of MFC cells that were treated with vehicle or M2-Exosome from wt or ApoE^-/-^ mouse. (f) Flow analysis for Apoptosis in MFC cell under the treatment of vehicle or M2-Exosome from wt or ApoE^-/-^ mouse. n.s., not significant by Student’s t-test (c).

**
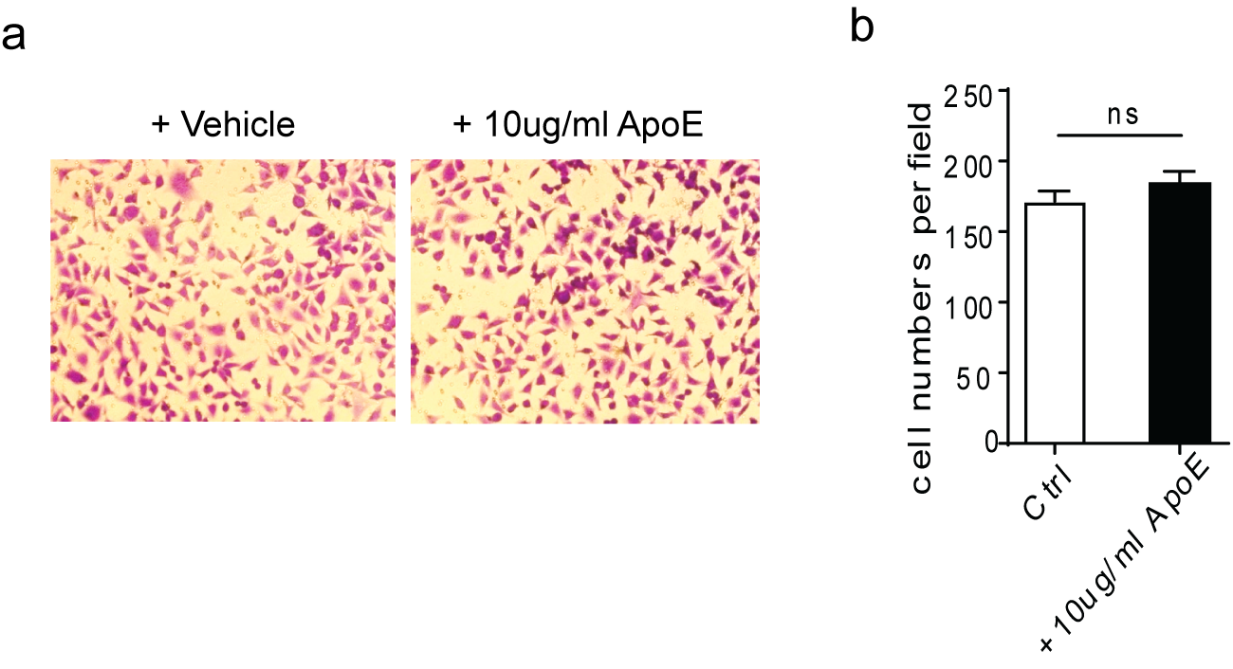
**

**Figure S5. The effect of recombinant ApoE on the migration of gastric cancer cells.**

(a) Migration assay of MFC cells treated with or without 10 ug/ml recombinant ApoE, (b) Quantification of migratory MFC cells with or without 10 ug/ml recombinant ApoE. Shown is the mean ± SEM of three independent experiments. n.s., not significant by Student’s t-test (b).

**
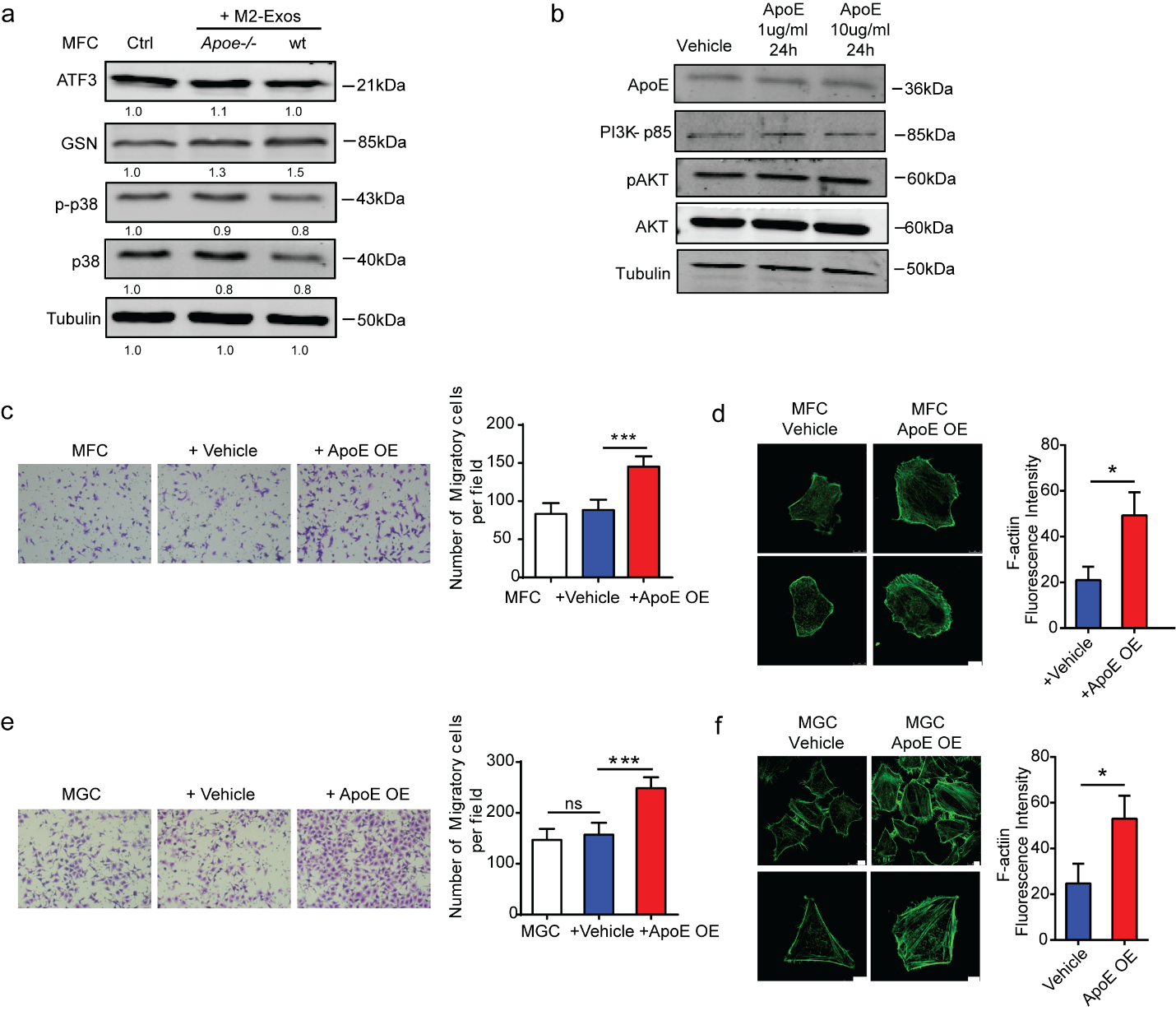
**

**Figure S6. Overexperssion ApoE induced aggressiveness of gastric cancer cells.**

(a) Western blot analysis of ATF3, GSN, p-p38, and p38, after MFC cells treated with M2-exosome of macrophage derived from ApoE^-/-^ or wt BM. Tubulin was used as the loading control. (b) Western blot analysis of PI3K- Akt signaling proteins after MFC cells were treated with recombinant ApoE for 24h. GAPDH was used as the loading control. (c) Migration assay of MFC cells transfected with ApoE overexpression plasmid or vehicle, with quantification of migratory MFC cells. (d) Representative immunofluorescence and quantification for actin staining (green) using phalloidin (scale bar, 10 µm) in MFC cells transfected with ApoE overexpression plasmid or vehicle. (e) Migration assay of MGC cells transfected with ApoE overexpression plasmid or vehicle, with quantification of migratory MGC cells. (f) Representative immunofluorescence and quantification for actin staining (green) using phalloidin (scale bar, 10 µm) in MGC cells transfected with ApoE overexpression plasmid or vehicle. Error bars represent mean ± s.e.m. **P* < 0.05, ****P* < 0.001; n.s., not significant; by one-way ANOVA with Dunnett’s multiple-comparison test (c, e) or Student’s t-test (d, f).

**Supplementary Table**

**Table S1:** The list of 19 solid tumors

| **Abbreviation** | **Full name of Tumor types** |
| --- | --- |
| BLCA | Urothelial bladder cancer |
| BRCA | Breast invasive carcinoma |
| CESC | Cervical squamous cell carcinoma and endocervical adnocarcinoma |
| CRC | Colorectal adenocarcinoma |
| GBM | Glioblastoma multiforme |
| HNSC | Head and neck squamous cell carcinoma |
| KICH | Kidney chromophobe |
| KIRC | Kidney renal clear cell carcinoma |
| KIRP | Kidney renal papillary cell carcinoma |
| LIHC | Liver hepatocellular carcinoma |
| LUAD | Lung adenocarcinoma |
| LUSC | Lung squamous cell carcinoma |
| OV | Ovarian serous cystadenocarcinoma |
| PAAD | Pancreatic ductal adenocarcinoma |
| PRAD | Prostate adenocarcinoma |
| SKCM | Skin cutaneous melanoma |
| STAD | Stomach adenocarcinoma |
| THCA | Thyroid carcinoma |
| UCEC | Uterine corpus endometrioid carcinoma |

**Table S2. The forward and reverse primer sequence were listed.**

| **Gene** | **FORWARD** | **REVERSE** |
| --- | --- | --- |
| M-ARG1 | 5-CAGAAGAATGGAAGAGTCAG | 5-CAGATATGCAGGGAGTCACC |
| M-INOS | 5-CTGCAGCACTTGGATCAGGAAC | 5-GGAGTAGCCTGTGTGCACCT |
| M-ApoE | 5-ACCGCTTCTGGGATTACCT | 5-TTCCGTCATAGTGTCCTCCA |
| M-IL-12 | 5-CCAAACCAGCACATTGAAGA | 5-CTACCAAGGCACAGGGTCAT |
| M-TNF- α | 5-ACGGCATGGATCTCAAAGAC | 5-GTGGGTGAGGAGCACGTAGT |
| M-CCL22 | 5-GCTCTCGTCCTTCTTGCTGT | 5-GGGTGACGGATGTAGTCCTG |
| M-TGF-β | 5-ATTCCTGGCGTTACCTTGG | 5-AGCCCTGTATTCCGTCTCCT |
| M-IL-10 | 5-GCCTTATCGGAAATGATCCA | 5-TGAGGGTCTTCAGCTTCTCAC |
| M-GAPDH | 5-AGGAGAGTGTTTCCTCGTCC | 5-GGCCTCACCCCATTTGATGT |
| H-ApoE | 5-CACTGTCTGAGCAGGTGCAG | 5-TCCAGTTCCGATTTGTAGGC |
| H-IL-12 | 5-TCAGTTTGGCCAGAAACCTC | 5-GTTTGTCTGGCCTTCTGGAG |
| H-CCL22 | 5-TCCTCGTCCTCCTTGCTGT | 5-GGTCCAGTAGAAGTGTTTCACCA |
| H-TGF-β | 5-CTGGCGATACCTCAGCAAC | 5-TAAGGCGAAAGCCCTCAAT |
| H-IL-10 | 5-CCAAGACCCAGACATCAAGG | 5-GCATTCTTCACCTGCTCCAC |
| H-TNF-α | 5-TTTCCGTGAAAACGGAGGCT | 5-CTAAGCAACCTTTATTTCTCGCC |
| H-IRF-4 | 5-GGAGAGCATTCAGCTTGCCT | 5-CGTAGCCCCTCAGGAAATGT |
| H-MRC1 | 5-GACGTGTGCACCTACCTCAA | 5-AGGACAGACCAGTACAATTCAG |
| H-GAPDH | 5-CTCTGCTCCTCCTGTTCGAC | 5-GCGCCCAATACGACCAAATC |

**Table S3: Antibody list for Western blot, IF, IHC and F**low cytometry.

| **Name** | **Host** | **Clone** | **Company** | **Dilution** |
| --- | --- | --- | --- | --- |
| CD68-PE | Mouse | Y1/82A | BD Pharmingen | 1:50(FC) |
| CD11b-V450 | Mouse | ICRF44 | BD Pharmingen | 1:50(FC) |
| CD163-PE | Mouse | GHI/61 | BD Pharmingen | 1:50(FC) |
| CD206-APC | Mouse | 19.2 | eBioscience | 1:50(FC) |
| CD206-APC | Rat | C068C2 | Biolegend | 1:50(FC) |
| CD80-PCy7 | Mouse | L307.4 | BD Pharmingen | 1:50(FC) |
| HLA-DR-V450 | Mouse | L243 | Biolegend | 1:50(FC) |
| F4/80-PE | Rat | BM8 | eBioscience | 1:50(FC) |
| CD11b-PCy5.5 | Rat | M1/70 | BD Pharmingen | 1:50(FC) |
| CD86-V450 | Rat | GL1 | BD Pharmingen | 1:50(FC) |
| CD63 | Rabbit | N/A | SBI | 1:1000(WB) |
| CD9 | Rabbit | N/A | SBI | 1:1000(WB) |
| CD81 | Rabbit | N/A | SBI | 1:1000(WB) |
| HSP70 | Rabbit | N/A | SBI | 1:1000(WB) |
| Apoe | Rabbit | EPR19392 | Abcam | 1:2000(WB,IHC) |
| N-cad | Rabbit | EPR1791-4 | Abcam | 1:1000(WB) |
| Vimentin | Rabbit | D21H3 | Cell Signaling | 1:1000(WB) |
| Snail | Rabbit | C15D3 | Cell Signaling | 1:1000(WB) |
| Twist | Rabbit | Polyclonal | Santa Cruz | 1:200(WB) |
| MMP-9 | Rabbit | Polyclonal | Millipore | 1:1000(WB) |
| MMP-2 | Rabbit | EP1183Y | Millipore | 1:1000(WB) |
| p-mTOR (Ser2448) | Rabbit | Polyclonal | Cell Signaling | 1:1000(WB) |
| mTOR | Rabbit | 7C10 | Cell Signaling | 1:1000(WB) |
| PI3K p85 | Rabbit | 19H8 | Cell Signaling | 1:1000(WB) |
| p-AKT (Thr308) | Rabbit | D25E6 | Cell Signaling | 1:1000(WB) |
| AKT | Rabbit | 11E7 | Cell Signaling | 1:1000(WB) |
| GSN | Mouse | 2C4 | Sigma-Aldrich | 1:5000(WB) |
| ATF-3 | Rabbit | Polyclonal | Santa Cruz | 1:200(WB) |
| p-P38 (Thr180/Tyr182) | Rabbit | 12F8 | Cell Signaling | 1:1000(WB) |
| P38 | Rabbit | D13E1 | Cell Signaling | 1:1000(WB) |
| Tubulin | Mouse | B512 | Sigma-Aldrich | 1:5000(WB) |
| CD68 | Mouse | KP1 | Abcam | 1:200(IHC) |
| CD163 | Rabbit | Polyclonal | Abcam | 1:200(IHC) |
| F4/80-Alexa Fluor647 | Rat | BM8 | BioLegend | 1:50(IF) |
| CD206-Alexa Fluor488 | Rat | C068C2 | Biolegend | 1:50(IF) |

**Table S4:** The overlap proteins were identified from M2-exosomes using density gradient ultracentrifugation and ExoQuick™ Extraction kit method by mass spectrometry.

| **Protein names** | **Relative signaling Value** | **Mol. weight [kDa]** |
| --- | --- | --- |
| Apolipoprotein E | 692805000 | 35.866 |
| Actin, cytoplasmic 2 | 292595000 | 41.792 |
| Alpha-2-macroglobulin | 167640000 | 165.85 |
| Haptoglobin | 84920500 | 38.752 |
| Fibronectin;Anastellin | 72963500 | 272.53 |
| Ferritin;Ferritin light chain 1;Ferritin light chain 2 | 61850500 | 20.756 |
| Pyruvate kinase isozymes M2 | 45154500 | 57.844 |
| Cathepsin B | 31055000 | 37.279 |
| Complement C1q subcomponent subunit C | 26019000 | 25.991 |
| Serum albumin | 23576000 | 68.692 |
| Triosephosphate isomerase | 19225450 | 32.191 |
| Macrophage metalloelastase | 17842200 | 54.97 |
| Histone H4 | 17001000 | 11.367 |
| Antithrombin-III | 16805500 | 52.003 |
| Alpha-enolase;Enolase | 16676500 | 47.14 |
| Tubulin alpha-1B chain;Tubulin alpha-1A chain;Tubulin alpha-1C chain;Tubulin alpha-3 chain | 15375350 | 50.179 |
| L-lactate dehydrogenase A chain | 13920000 | 39.758 |
| Keratin, type II cytoskeletal 2 oral | 13029000 | 62.844 |
| Creatine kinase M-type | 12819000 | 43.044 |
| Fructose-bisphosphate aldolase;Fructose-bisphosphate aldolase A | 12410850 | 45.12 |
| Heat shock cognate 71 kDa protein;Heat shock-related 70 kDa protein 2 | 11461650 | 70.87 |
| Heat shock protein HSP 90-alpha | 11455950 | 84.787 |
| Coagulation factor X | 10615500 | 55.255 |
| Gelsolin, isoform | 10417000 | 80.848 |
| Profilin-1;Profilin | 10251500 | 15.057 |
| Elongation factor 1-alpha 1;Elongation factor 1-alpha 2 | 9822000 | 50.113 |
| Fibrinogen gamma chain | 9637050 | 50.349 |
| Thrombospondin-4 | 9441400 | 106.37 |
| Glutathione peroxidase 3 | 7202450 | 25.442 |
| 14-3-3 protein zeta/delta | 6630600 | 27.771 |
| Serum paraoxonase/arylesterase 1 | 6360400 | 39.565 |
| Actin, cytoplasmic 1 (Fragment) | 6006000 | 29.442 |
| Apolipoprotein B-100 | 5987000 | 509.43 |
| Adenosylhomocysteinase | 5882600 | 47.688 |
| Galectin-3-binding protein | 5601350 | 64.49 |
| Glyceraldehyde-3-phosphate dehydrogenase | 5387500 | 38.653 |
| Peroxiredoxin-4 | 4451450 | 31.052 |
| Lipoprotein lipase | 4130650 | 53.109 |
| Isocitrate dehydrogenase [NADP] | 3810850 | 47.545 |
| Polyubiquitin-C;Ubiquitin;Ubiquitin-related 1;Ubiquitin-related 2;Polyubiquitin-B;Ubiquitin | 3763700 | 82.549 |
| Heat shock protein HSP 90-beta | 3612750 | 83.28 |
| Vimentin | 3026250 | 53.687 |
| Complement C3 | 3009750 | 186.48 |
| Cartilage oligomeric matrix protein | 2864450 | 82.802 |
| 14-3-3 protein gamma;14-3-3 protein gamma, N-terminally processed;14-3-3 protein eta;14-3-3 protein beta/alpha;14-3-3 protein beta/alpha, N-terminally processed | 2772950 | 28.302 |
| Serpin H1 | 2739550 | 46.533 |
| Keratin, type II cytoskeletal 72 | 2411050 | 58.224 |
| 78 kDa glucose-regulated protein | 2284795 | 72.421 |
| Actin, cytoplasmic 1;Actin, cytoplasmic 1, N-terminally processed | 2242600 | 41.736 |
| Phosphatidylethanolamine-binding protein 1 | 1889050 | 23.035 |
| Clathrin heavy chain 1 | 1859370 | 192.48 |
| Phosphoglycerate kinase 1;Phosphoglycerate kinase 2 | 1773365 | 44.55 |
| Peroxiredoxin-6 | 1627750 | 25.07 |
| Phosphoglycerate mutase 1 | 1350450 | 28.832 |
| Adenylyl cyclase-associated protein 1 | 1267050 | 51.564 |
| Thrombospondin-1 | 1030600 | 129.72 |
| Alpha-actinin-4;Alpha-actinin-1 | 975465 | 104.98 |
| L-lactate dehydrogenase B chain | 784400 | 36.572 |
| Elongation factor 2 | 726820 | 95.313 |
| Basement membrane-specific heparan sulfate proteoglycan core protein;Endorepellin;LG3 peptide | 477765 | 469.78 |
| Myosin-9 | 255615 | 226.37 |
| Phosphorylase;Glycogen phosphorylase, liver form;Glycogen phosphorylase, brain form;Glycogen phosphorylase, muscle form | 207385 | 97.456 |
| Contactin-1 | 192170 | 113.39 |
| Talin-1 | 189980 | 272.13 |
| Heat shock 70 kDa protein 1B;Heat shock 70 kDa protein 1A | 184215 | 70.175 |
| Filamin-A | 179585 | 281.22 |
| Low-density lipoprotein receptor-related protein 1 | 54340 | 504.77 |
| Fructose-bisphosphate aldolase B | 0 | 39.521 |
| Hemoglobin subunit epsilon-Y2 | 0 | 16.136 |
| Murinoglobulin-1;Murinoglobulin-2 | 0 | 165.3 |
| Protein disulfide-isomerase A3;Thioredoxin | 0 | 56.678 |
| Sacsin | 0 | 520.69 |
